# Discrimination of leaf diseases in Maize/Soybean intercropping system based on hyperspectral imaging

**DOI:** 10.3389/fpls.2024.1434163

**Published:** 2024-12-09

**Authors:** Xin Liu, Kaixin Meng, Kaixing Zhang, Wujie Yang, Jiutao Yang, Lingyang Feng, Haoran Gong, Chang’an Zhou

**Affiliations:** ^1^ Peking University Institute of Advanced Agricultural Sciences, Shandong Laboratory of Advanced Agricultural Sciences, Weifang, Shandong, China; ^2^ College of Agronomy, College of Mechanical and Electronic Engineering, Shandong Agricultural University, Taian, Shandong, China; ^3^ Crop Technology Promotion Department 1, Shandong Agricultural Technology Extension Center, Jinan, Shandong, China

**Keywords:** hyperspectral feature extraction, crop disease detection, machine learning, intelligent optimization, non-invasive identification

## Abstract

In order to achieve precise discrimination of leaf diseases in the Maize/Soybean intercropping system, i.e. leaf spot disease, rust disease, mixed leaf diseases, this study utilized hyperspectral imaging and deep learning algorithms for the classification of diseased leaves of maize and soybean. In the experiments, hyperspectral imaging equipment was used to collect hyperspectral images of leaves, and the regions of interest were extracted within the spectral range of 400 to 1000 nm. These regions included one or more infected areas on the leaves to obtain hyperspectral data. This approach aimed to enhance the accurate discrimination of different types of diseases, providing more effective technical support for the detection and control of crop diseases. The preprocessing of hyperspectral data involved four methods: Savitzky-Golay (SG), Standard Normal Variate (SNV), Multiplicative Scatter Correction (MSC) and 1st Derivative (1st Der). The 1st Der was found to be the optimal preprocessing method for hyperspectral data of maize and soybean diseases. Competitive Adaptive Reweighted Sampling (CARS), Successive Projections Algorithm (SPA) and Principal Component Analysis (PCA) were employed for feature extraction on the optimal preprocessed data. The Support Vector Machines (SVM), Bidirectional Long Short-Term Memory Network (BiLSTM) and Dung Beetle Optimization-Bidirectional Long Short-Term Memory Network (DBO-BiLSTM) were established for the discrimination of maize and soybean diseases. Comparative analysis indicated that, in the classification of maize and soybean diseases, the DBO-BiLSTM model based on the CARS extraction method (1st Der-CARS-DBO-BiLSTM) demonstrated the highest classification rate, reaching 98.7% on the test set. The research findings suggest that integrating hyperspectral imaging with both traditional and deep learning methods is a viable and effective approach for classifying diseases in the intercropping model of maize and soybean. These results offer a novel method and a theoretical foundation for the non-invasive, precise, and efficient identification of diseases in the intercropping model of maize and soybean, carrying positive implications for agricultural production.

## 1 Introduction

Corn (Zea mays L.), commonly known as maize, boasts cultivation acreage and yield surpassed only by wheat and rice, ranking third among global cereal crops. Its yield per unit area claims the top spot worldwide (Sun et al., 2021). As one of primary grain crops, corn plays a pivotal role not only as a crucial feedstock for the livestock industry but also as an industrial raw material (Vapnik and Cortes, 1995). Its quality and yield significantly impact the development of national economy. Diseases represent a critical factor influencing corn production and quality. Soybean (Glycine max (L.) Merr.), a perennial herbaceous plant belonging to the legume family and Glycine genus, originated in China and is extensively cultivated nationwide, as well as globally. As a vital grain crop in China with a long cultivation history, soybean is predominantly grown in the northeast regions of China (Wu et al., 2022). The seeds of soybean is rich in plant proteins, with a content ranging from 35% to 40%. Soybean, being an agricultural product, have garnered widespread attention worldwide.

As the population continues to increase and the overall demand for agricultural economic development grows, enhancing the yield and quality of maize and soybean has become increasingly urgent (Xiong et al., 2016). Traditional agricultural planting methods and technologies are gradually falling behind in terms of crop yield, necessitating the introduction and application of new planting techniques (Yuan et al., 2021). Intercropping, as a comprehensive planting model that integrates green environmental practices, resource optimization, and balanced development, has successfully achieved efficient utilization of resources such as land, water, fertilizers, light, and space (Jinchi et al., 2005). It stands out as a crucial planting model for building resource-efficient and environmentally friendly ecological agriculture. The intercropping model of maize and soybean significantly improves the utilization of planting space while reducing the input of planting and labor costs. This not only promotes an increase in crop yield but also enhances the economic returns of crops.

With the promotion of the intercropping mode of maize and soybean, one of the primary factors affecting yield during the planting process is pests and diseases (Yuan P. et al., 2021) Therefore, the ensuing challenge is how to address the issue of pests and diseases specifically and effectively in maize and soybean. To tackle this problem with greater recall and efficiency, the discrimination and detection of the types of pests and diseases in the intercropping systems become particularly crucial (Zhang F. et al., 2023). Currently, Plant disease detection commonly relies on two primary methods: manual experience judgment and physicochemical testing. However, both methods have their limitations. Manual experience judgment is susceptible to subjective and objective factors such as weather conditions, health status, and emotions, potentially leading to misjudgments (Wu et al., 2023). On the other hand, while physicochemical testing yields more accurate results, it demands higher operational skills and suffers from drawbacks like complexity, sample destructiveness, and poor timeliness. These shortcomings significantly restrict the application of both methods in practical production, necessitating the search for more applicable detection techniques (Razzaq et al., 2019).

Currently, non-destructive measurement technologies are rapidly advancing and finding applications in various aspects of agriculture (Fu et al., 2019). Hyperspectral imaging is a typical application in this field, with applications in both large-scale remote sensing and precise spectral imaging. In large-scale remote sensing, this technology is used to measure the yield, soil nutrient conditions, and drought levels of extensive crop areas (Zhang F. et al., 2023). In the domain of precise spectral imaging, hyperspectral imaging enables detailed analysis of plant conditions and provides comprehensive diagnostics for diseases. This opens up new avenues for finding more accurate and efficient methods for plant disease detection (Hao et al., 2017; Yu et al., 2019).

Hyperspectral imaging has been extensively developed in rapid and non-destructive plant disease detection (Shu et al., 2022; Jung et al., 2022). For instance, it has been employed to detect beech leaf disease using SVM and Random Forest algorithms (RF) (Fearer et al., 2022) and to identify Tobacco mosaic virus (TMV) and Potato virus Y disease (PVY) (Chen et al., 2023). However, due to the complex redundancy of hyperspectral data, the accuracy of direct detection using classification models still requires improvement (Wang F. et al., 2019). Therefore, many researchers currently adopt preprocessing and feature extraction of hyperspectral data to enhance its accuracy. For example, multivariate scatter correction has been utilized to preprocess the rice bacterial stripe disease spectra, achieving a maximum classification accuracy of 95.24% with an RF classifier (Yuan P. et al., 2021). Similarly, various preprocessing methods have been applied to enhance the stability of the citrus disease leaf detection model (Wu et al., 2021). To further simplify hyperspectral data, spectral feature extraction is performed on processed data, such as constructing a model for identifying cotton pest-infested leaves using Principal Component Analysis (PCA) combined with SVM (Wang et al., 2019b). As machine learning continues to evolve, intelligent optimization algorithms are introduced to enhance the performance of classification models. For example, Genetic Algorithm (GA), Particle Swarm Optimization (PSO), and Grey Wolf Optimizer (GWO) have been employed to optimize an SVM classification model for the categorization of 27 Oolong tea varieties, achieving an optimized classification rate of 99.91% (Cao et al., 2023).In addition to integrating traditional learning methods with hyperspectral data, numerous studies explore combining deep learning algorithms with hyperspectral imaging for non-destructive plant disease detection. Examples include the detection of PVY infection in potato plants (Polder et al., 2019), identification of cotton aphid disease (Yan et al., 2021), detection of maize diseases (Fu et al., 2022), early diagnosis of strawberry gray mold disease (Jung et al., 2022), and accurate identification of maize varieties (Zhang F. et al., 2023). Researchers also used SVM, Logistic Regression (LR), and Convolutional Neural Networks (CNN) to construct classification models based on different levels of fusion, and the results showed that the CNN model outperformed the SVM and LR models (Feng et al., 2020). Collectively, these studies highlight the significant effectiveness of combining hyperspectral imaging with both traditional and deep learning approaches for crop disease detection. In addition, currently, most studies focus on the identification and detection of single-crop and single-pathogen or a limited number of pathogens in monoculture systems, such as maize leaf spot disease in monoculture (Wang et al., 2019a; Xu et al., 2020) and soybean mildew (Zhang F. et al., 2023). Additionally, studies address single-crop, dual-disease scenarios like soybean**’**s angular leaf spot and bacterial spot (Liu et al., 2023), and cotton**’**s aphid and red spider mite pests. However, there is relatively less research on intercropping systems involving multiple crops and multiple diseases (Yuan R. et al., 2021). This is mainly due to the need for the model to first identify leaf types before detecting diseases on the leaves,making the identification process more complex compared to monoculture systems (Zhang Y. et al., 2023). Additionally, the number of disease types increases in intercropping systems compared to monoculture. Therefore, the focus of this study is to identify different disease types in intercropping patterns of maize and soybean based on hyperspectral identification and to be able to discriminate the diseases under different environmental factors.

This paper addresses the problem of fewer research on detection of multiple species in intercropping mode. Based on hyperspectral imaging technology, this paper established a classification model for multi-crops and diseases in the maize/soybean intercropping system in order to improve the yield and quality of the two crops and to provide a basic method for the detection of multi-crop diseases in subsequent intercropping. The main research includes:

1. Multiple preprocessing methods and feature extraction techniques are used to process hyperspectral data, and the set is divided into a training set and a test set, establishing multiple classification and detection models, and selecting the combination of methods with optimal classification performance through comparison.
2. Optimizing the BiLSTM neural network model using the DBO algorithm to find the best parameter settings. The DBO-BiLSTM leaf disease classification model was established, which effectively improved the accuracy of disease classification and detection in the corn/soybean intercropping system.
3. A classification model capable of identifying various types of crops and diseases in the intercropping model was established, thus improving the efficiency of disease classification for different crops. This lays a solid foundation for targeted management of crop diseases.

## 2 Materials and methods

**Figure 1:**
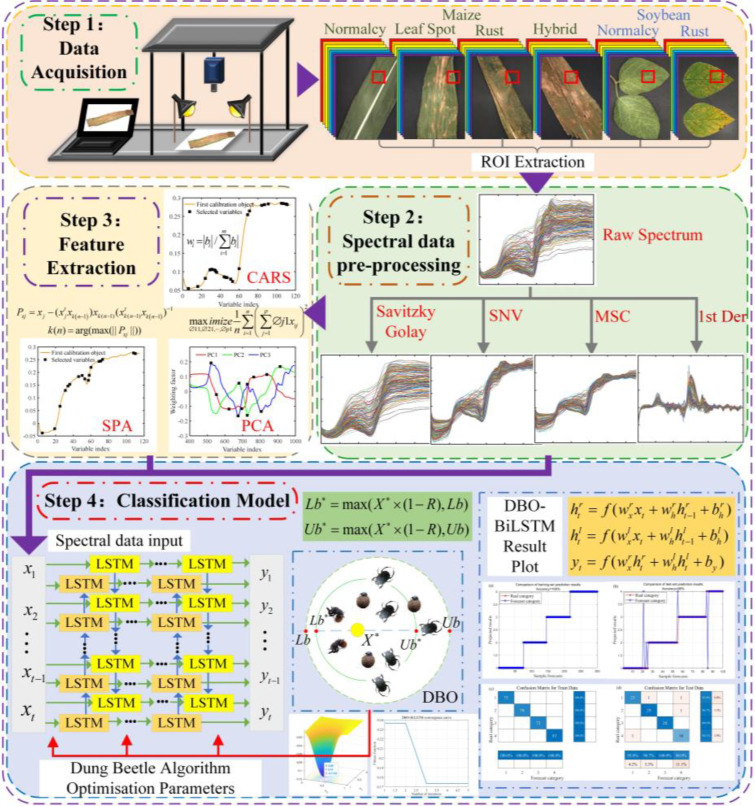
Framework diagram.

The research framework of this paper consists of four main steps, the framework diagram is shown in **Figure 1**. The first step involves sample preparation and data acquisition. In the second step, four preprocessing methods—SG, SNV, MSC, and 1st Der—are employed to preprocess the raw hyperspectral data. The processed data is then fed into the model to obtain the optimal preprocessing method. The third step involves employing CARS, SPA, and PCA methods for feature selection on the preprocessed data, further enhancing the model’s accuracy. The fourth step encompasses establishing SVM, BiLSTM, and DBO-BiLSTM models using the feature-extracted hyperspectral data. The model combination with the highest classification accuracy is selected through comparative analysis.

### 2.1 Test materials and hyperspectral data acquisition

**Figure 2:**
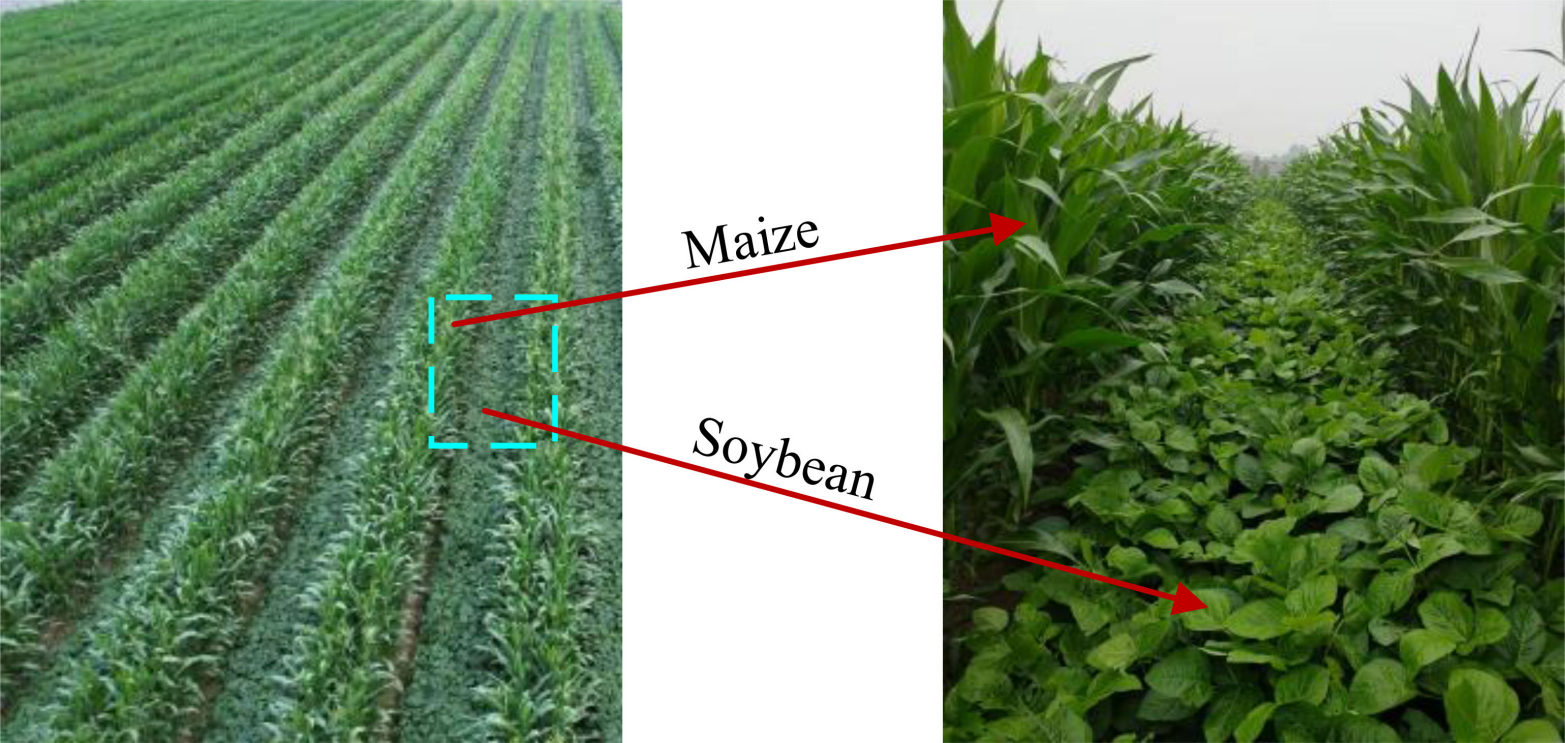
Maize-Soybean intercropped fields.

**Figure 3:**
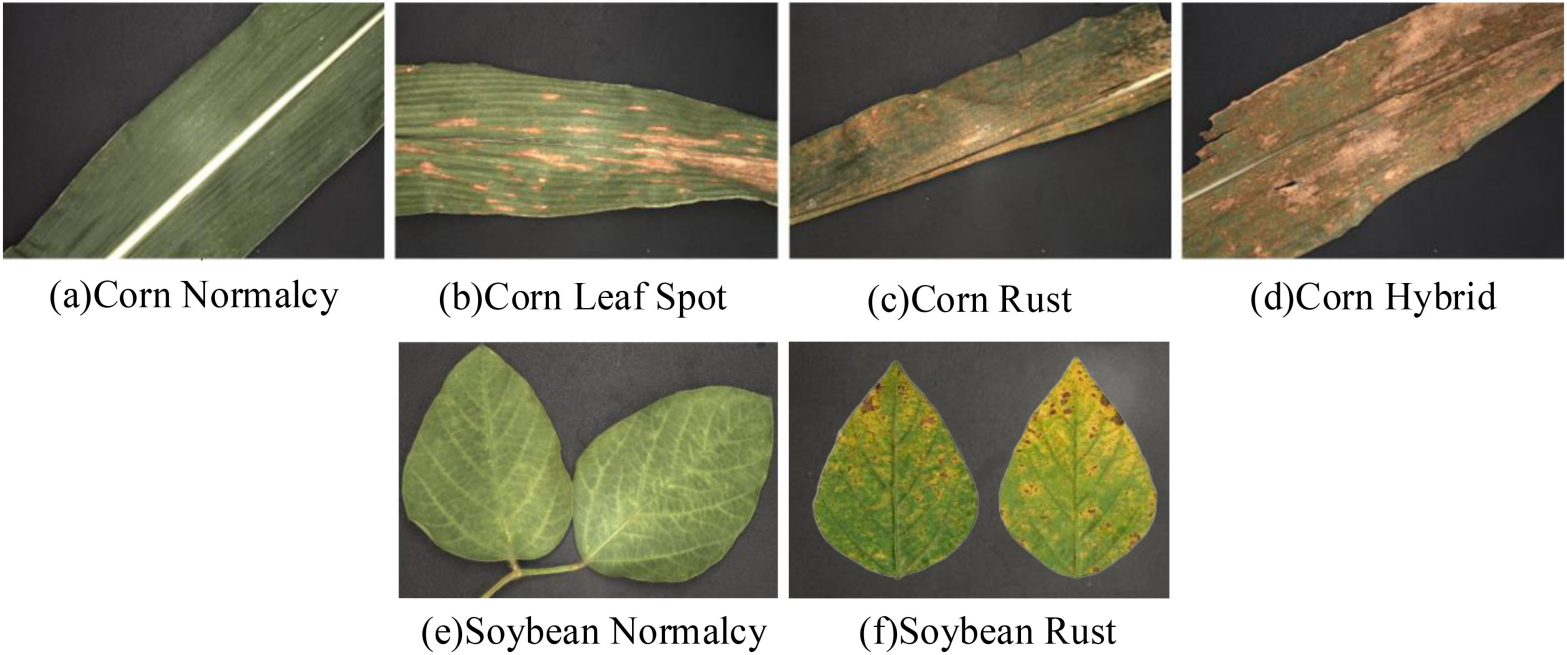
Sample image of a portion of a diseased leaf. **(A)** Corn Normalcy; **(B)** Corn Leaf Spot; **(C)** Corn Rust; **(D)** Corn Hybrid; **(E)** Soybean Normalcy; **(F)** Soybean Rust.

The samples for this experiment were acquired on September 13, 2023, from maize-soybean intercropping fields in Fei Cheng City, Tai’an City, Shandong Province, China, the maize-soybean intercropping field is shown in **Figure 2**. The acquired samples include maize leaves with leaf spot disease as shown in **Figure 3B**, rust-infested maize leaves as shown in **Figure 3C**, rust-infested soybean leaves as shown in **Figure 3F**, as well as normal maize leaves as shown in **Figure 3A**, and normal soybean leaves as shown in **Figure 3E**. In addition, the samples also included a mixture of maize diseases as shown in **Figure 3D**. After collection, the samples were placed in sealed bags and transported to the laboratory for processing immediately. Following the removal of surface dust, hyperspectral images were acquired using a hyperspectral spectrometer.

**Figure 4:**
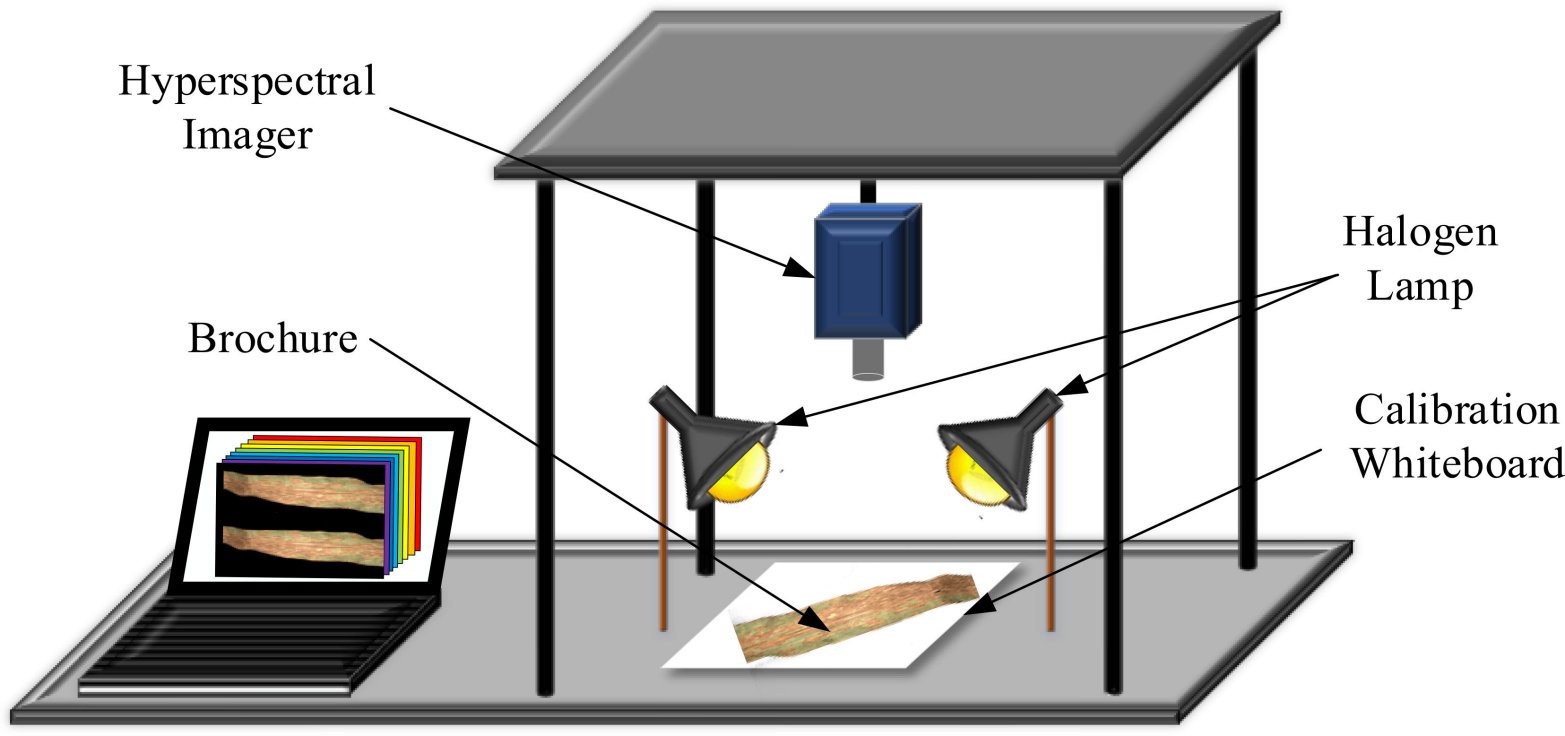
Hyperspectral data acquisition system.

To obtain hyperspectral images of the samples, a hyperspectral data acquisition system based on a hyperspectral spectrometer was established. The system comprises the following main components: hyperspectral imaging spectrometer (Brand U.S.A. SOC Model SOC710VP^®^), lenses, two symmetrically distributed halogen linear light sources, a full-diffuse polytetrafluoroethylene calibration whiteboard, a tripod, an experimental platform, a dark box, and computer equipment, as depicted in **Figure 4**.

When obtaining hyperspectral images, the process involved securing the hyperspectral spectrometer on a tripod and ensuring the lens was perpendicular to the sample, inserted into a dark box to ensure complete imaging of the maize and soybean leaf samples. Simultaneously, it was ensured that the halogen linear light source was the sole light source during collection, focusing the light onto the leaves. The hyperspectral spectrometer was preheated for 30 minutes until both the current and intensity of the halogen linear light source were stabilized. Finally, the relevant acquisition parameters were set using the Spec View software. To mitigate the impact of the uneven distribution of the halogen linear light source and eliminate noise caused by the dark current of the hyperspectral spectrometer, black and white correction was applied to the acquired hyperspectral images (Chi et al., 2021; Qu and Liu, 2017). The reflection correction formula is articulated as follows:


(1) $$R_{ci}=\frac{Sample_{ci}-dark_{ci}}{White_{ci}-dark_{ci}}$$

where $R_{ci}$ , $Sample_{ci}$ , $dark_{ci}$ , and $White_{ci}$ represent the calibrated hyperspectral image, the uncalibrated hyperspectral image, the reflectance intensity of the blackboard reference, and the reflectance intensity of the standard whiteboard reference, respectively.

This study collected hyperspectral images of 80 maize leaves and 40 soybean leaves. In the maize leaf group, there were 20 healthy leaves, 20 leaves with leaf spot disease, 20 leaves with rust disease, and 20 leaves with both diseases simultaneously. In the soybean group, there were 20 healthy leaves and 20 leaves with rust disease. ENVI 5.3 software was used to select regions of interest (ROIs) containing infected areas. Some leaves had multiple lesions, so it was ensured that only one type of lesion was present in each ROI. For instance, when selecting ROIs for leaves with leaf spot disease, regions with only leaf spots were chosen, and for mixed leaves, regions with both diseases were selected. The average spectrum of each ROI was considered as an individual sample. As the average spectrum was used for ROI selection, there was no limitation on the size of the ROIs. Following this approach, 400 maize data samples and 200 soybean data samples were obtained. Specifically, each type of maize and soybean leaf disease obtained 100 samples.

### 2.2 Hyperspectral data preprocessing

The initially collected spectra exhibit diverse noise interference and redundant information, posing a substantial impact on the accuracy of established model. Various methods have been employed to mitigate the influence of noise, spectral drift, and other interferences (Esquerre et al., 2012; Silalahi et al., 2018; Wu et al., 2019). In this investigation, the initial hyperspectral data underwent preprocessing utilizing four techniques: SG, MSC, SNV, and 1st Der.

### 2.3 Spectral data feature extraction

Critical spectral identification information is concentrated within specific feature wavelengths. The extraction of these feature wavelengths from the preprocessed full spectral bands diminishes data dimensionality, alleviates computational load, and amplifies the pace of model development and classification accuracy.

The Competitive Adaptive Reweighted Sampling (CARS) algorithm, introduced by Li et al., serves as a feature variable selection method (Li et al., 2014). The combination of Monte Carlo sampling and partial least squares regression modeling is an important aspect of this algorithm, mirroring the concept of “survival of the fittest” from Darwinian theory (Wang et al., 2021). In the CARS algorithm, adaptive weighted sampling is iteratively performed, retaining points with more significant absolute weight coefficients from the PLS model to form a new subset while discarding points with smaller weights. Subsequently, a new PLS model is established based on this updated subset. After multiple iterations, the algorithm identifies the wavelengths from the subset with the smallest Root Mean Square Error of Cross-Validation (RMSECV) as the feature wavelengths (Shao et al., 2021; Xu et al., 2022; Tian et al., 2022).

The Successive Projections Algorithm (SPA) is a widely employed technique for selecting feature bands, renowned for its resilience against interference (Shi et al., 2021; He et al., 2022). SPA, a variable selection method for multiple linear regression, utilizes an iterative, forward-selection-based approach. The algorithm computes the projection of variables onto the unselected variables through systematic iterations. Consequently, the wavelengths chosen as candidates for selection are those associated with the most significant projection vectors (Yuan P. et al., 2021; Meng et al., 2021).

Principal Component Analysis (PCA), also referred to as the Hotelling transform or K-L transform, has been widely utilized for extracting characteristic wavelengths from multi-band hyperspectral data. PCA involves transforming strongly correlated n-dimensional original variables into k-dimensional new variables that are both representative and uncorrelated, achieved through an orthogonal transformation. These k-dimensional variables are organized based on their contribution rate, from highest to lowest, facilitating the extraction of several mutually uncorrelated and representative bands from the hundreds of spectral bands. This method aims to address the issue of spectral data correlation, allowing the extraction of characteristic wavelengths while retaining the original spectral information as much as possible (Tian et al., 2020; Feng L. et al., 2020).

This study employs three methods for feature wavelength extraction: CARS, SPA and PCA.

### 2.4 Model establishment

#### 2.4.1 Bidirectional long short-term memory network

**Figure 5:**
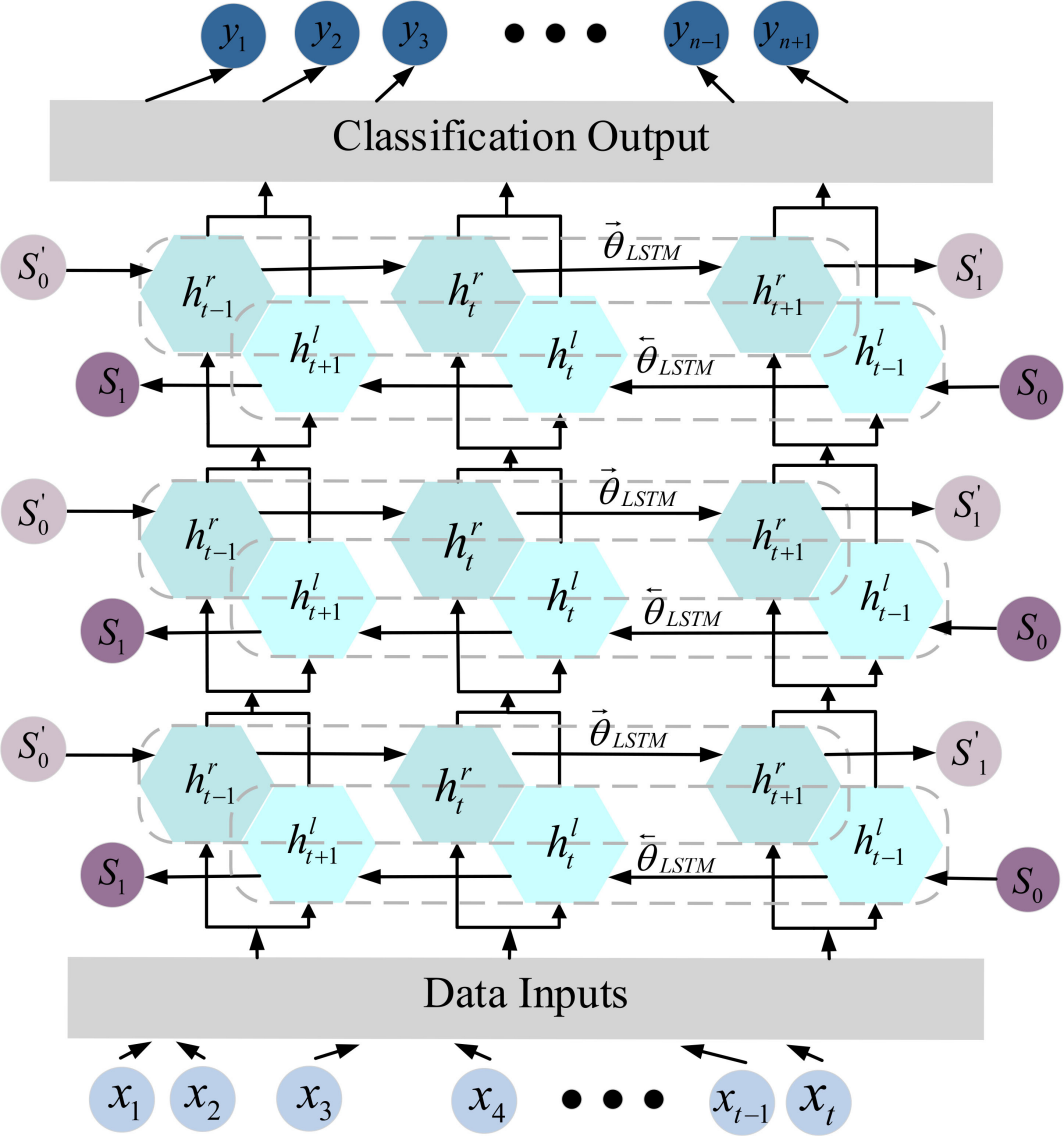
BiLSTM single neuron calculation process.

The Bidirectional Long-Term Memory (BiLSTM) represents an enhanced version of the Long Short-Term Memory (LSTM) network. In contrast to the conventional LSTM, which exclusively extracts sequential forward information, potentially overlooking valuable backward information, the BiLSTM network integrates both forward and backward outputs at each time step. This architectural improvement proves more efficient in capturing correlations between preceding and subsequent data points, thereby augmenting the model’s capacity to extract sequential information (Liu et al., 2020; Shi et al., 2023). The specific structural details are illustrated in **Figure 5**.

As shown in **Figure 5**, data enters into BiLSTM through the input layer. From left to right $\left(S_{0}^{'}\to S_{1}^{'}\right)$ the data processed is the forward LSTM layer, performing forward computation to obtain $h_{t}^{r}$ . Simultaneously, instead processing the data from right to left $\left(S_{0}\to S_{1}\right)$ is the backward LSTM layer, conducting backward analysis to get $h_{t}^{l}$ . After processing all time sequences, the hidden states of the two LSTM layers are concatenated, resulting in the final output $y_{t}$ for the BiLSTM model.

The calculation formula is as follows:


(2) $$\begin{array}{l} h_{t}^{r}=f(w_{x}^{r}x_{t}+w_{h}^{r}h_{t-1}^{r}+b_{h}^{r})\\ h_{t}^{l}=f(w_{x}^{l}x_{t}+w_{h}^{l}h_{t-1}^{l}+b_{h}^{l})\\ y_{t}=f(w_{x}^{r}h_{t}^{r}+w_{h}^{l}h_{t}^{l}+b_{y}) \end{array}$$

where $h_{t}^{r}$ and $h_{t}^{l}$ represent the hidden states of the forward and backward LSTM layers, respectively; $y_{t}$ is the current output of the BiLSTM; *f*, *w*, and *b* correspond to the activation function, weights, and biases, respectively.

#### 2.4.2 DBO-BiLSTM model

Due to the inclusion of multiple network parameters in the BiLSTM network, it is challenging to set the optimal parameters for the classification of maize and soybean diseases. Therefore, an optimization algorithm is needed to search for the best parameter values. However, simple optimization algorithms may face limitations in finding the optimal parameters. In this regard, this paper introduces the firefly algorithm to optimize the five parameters of the bidirectional long short-term memory network. These five parameters are the number of hidden units, maximum training epochs, mini-batch size, initial learning rate, and L2 regularization parameter.

The Dung Beetle Optimization (DBO) algorithm is an innovative swarm intelligence optimization technique inspired by the collective behaviors observed in dung beetle populations (Xue and Shen, 2023). These behaviors encompass various activities such as rolling, dancing, foraging, breeding, and stealing. Unlike traditional algorithms like Particle Swarm Optimization and Genetic Algorithms, the DBO algorithm introduces specific survival tasks within the dung beetle population. The population is categorized into four types of dung beetles: rollers, breeders (involved in breeding balls), small dung beetles, and thieves (Razzaq et al., 2019).

The rolling dung beetle is updated in position as it rolls, and the rolling mathematical model can be expressed as:


(3) $$\begin{array}{c} x_{i}(t+1)=x_{i}(t)+a\times k\times x_{i}(t-1)+b\times \text{Δ}x\\ \text{Δ}x=\left|x_{i}(t)-X^{W}\right| \end{array}$$

where *t* represents the current iteration count, $x_{i}$ denotes the position information of the *i* dung beetle at the *i* iteration, *k* ∈ (0,0.2] stands for the constant of deviation, *b* is a constant within the range (0, 1), *a* is a natural coefficient assigned either -1 or 1, $X^{W}$ represents the global worst position, and Δx is used to simulate variations in light intensity.

**Figure 6:**
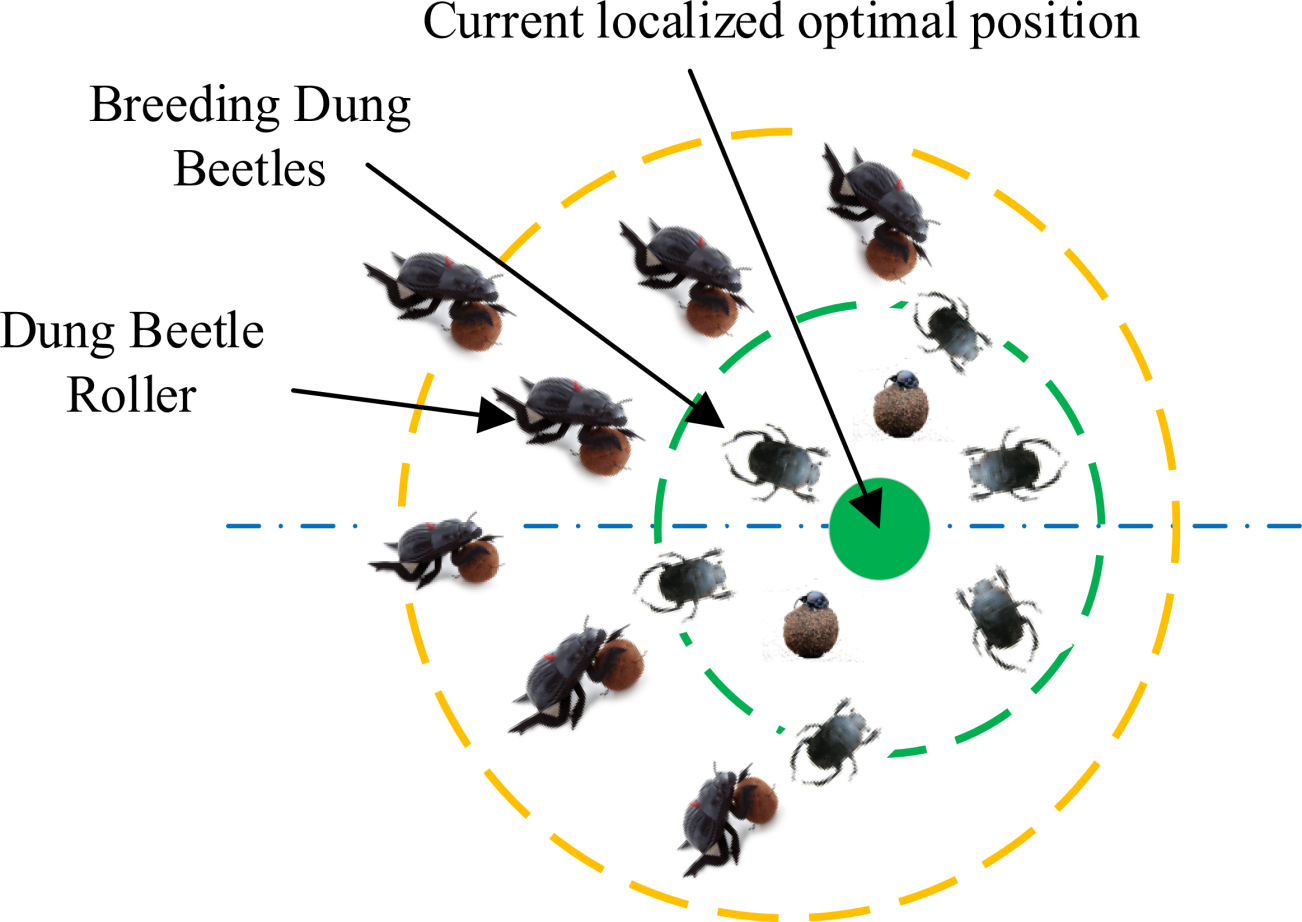
Conceptual model of the boundary selection strategy.


The breeding dung beetles select suitable areas for egg-laying based on the dung balls. Breeding dung beetle spawning areas in this process resembles a boundary selection strategy. The strategy is shown in **Figure 6**, and its mathematical model is as follows:

(4) $$\begin{array}{l} Lb^{*}=\max(X^{*}\times (1-R),Lb)\\ Ub^{*}=\max(X^{*}\times (1-R),Ub) \end{array}$$

where $X^{*}$ represents the current local best position, $Lb^{*}$ and $Ub^{*}$ signify the lower and upper bounds of the oviposition area, while Lb and Ub respectively denote the lower and upper limits of the optimization problem.

Selection of optimal spawning areas based on a boundary selection strategy, the female dung beetles will opt to lay their breeding balls within this designated region. Equation 3 distinctly illustrates that the boundaries of the oviposition area are dynamically variable, primarily determined by the *R-value*. Therefore, the position of the breeding balls during the iterative process is also dynamic, demonstrated by the following iteration:


(5) $$B_{i}(t+1)=X^{*}+b_{1}\times (B_{i}(t)-Lb^{*})+b_{2}\times (B_{i}(t)-Ub^{*})$$

where $B_{i}(t)$ refers to points of the compass of the *i* breeding ball at the *t* iteration, $b_{1}$ and $b_{2}$ r represent two random vectors of disjoint size and belonging to the 1×D , with *D* representing the dimension of the optimization problem.

After breeding, newborn juvenile dung beetles similarly crawl out of the ground in search of dung balls, guided by optimal foraging areas. This simulates the natural foraging process of these beetles in their habitat. The boundaries of the optimal foraging area are defined as follows:


(6) $$\begin{array}{l} Lb^{b}=\max(X^{b}\times (1-R),Lb)\\ Ub^{b}=\max(X^{b}\times (1-R),Ub) \end{array}$$

where $X^{b}$ represents the global best position, $Lb^{b}$ and $Ub^{b}$ denote the optimal foraging area’s lower and upper limits. The position of the dung beetle is updated with the following equation:


(7) $$x_{i}(t+1)=x_{i}(t)+C_{1}\times (x_{i}(t)-Lb^{b})+C_{2}\times (x_{i}(t)-Ub^{b})$$

where $x_{i}(t)$ indicates the position information of the i dung beetle at the *t* iteration, $C_{1}$ represents a random number following a normal distribution, and $C_{2}$ is a random number with values in the range (0,1).

The stealing dung beetles search for the best food source and conduct theft. From Equation 6, $X^{b}$ represents the global best position, which denotes the best food source. Hence, during the stealing process, the position information of the stealing dung beetles will be updated as follows:


(8) $$x_{i}(t+1)=x^{b}+S\times u\times \left(\left|x_{i}(t)-X^{*}\right|+\left|x_{i}(t)-X^{b}\right|\right)$$

where $x_{i}(t)$ represents the orientation of the *i* stealing dung beetle at the *t* iteration, *u* is a random vector of size 1×D obeying a Gaussian distribution, and *S* denotes a fixed variable.

Following the preprocessing of raw data, dimensionality reduction through feature extraction algorithms, and parameter optimization for the BiLSTM model using the Dung Beetle Optimization algorithm, the optimal hyperparameters are implemented in the model, giving rise to the DBO-BiLSTM model. The positions are updated through four dung beetle behaviors: rolling dung, breeding, foraging, and theft. Ultimately, the best solution and its fitness value are determined. The optimal solution, representing the most effective hyperparameter configuration for the bidirectional BiLSTM model, is then validated on test set and applied to real-world data analysis tasks.

## 3 Results and analysis

### 3.1 Hyperspectral data preprocessing

#### 3.1.1 Preprocessing results and analyses

**Figure 7:**
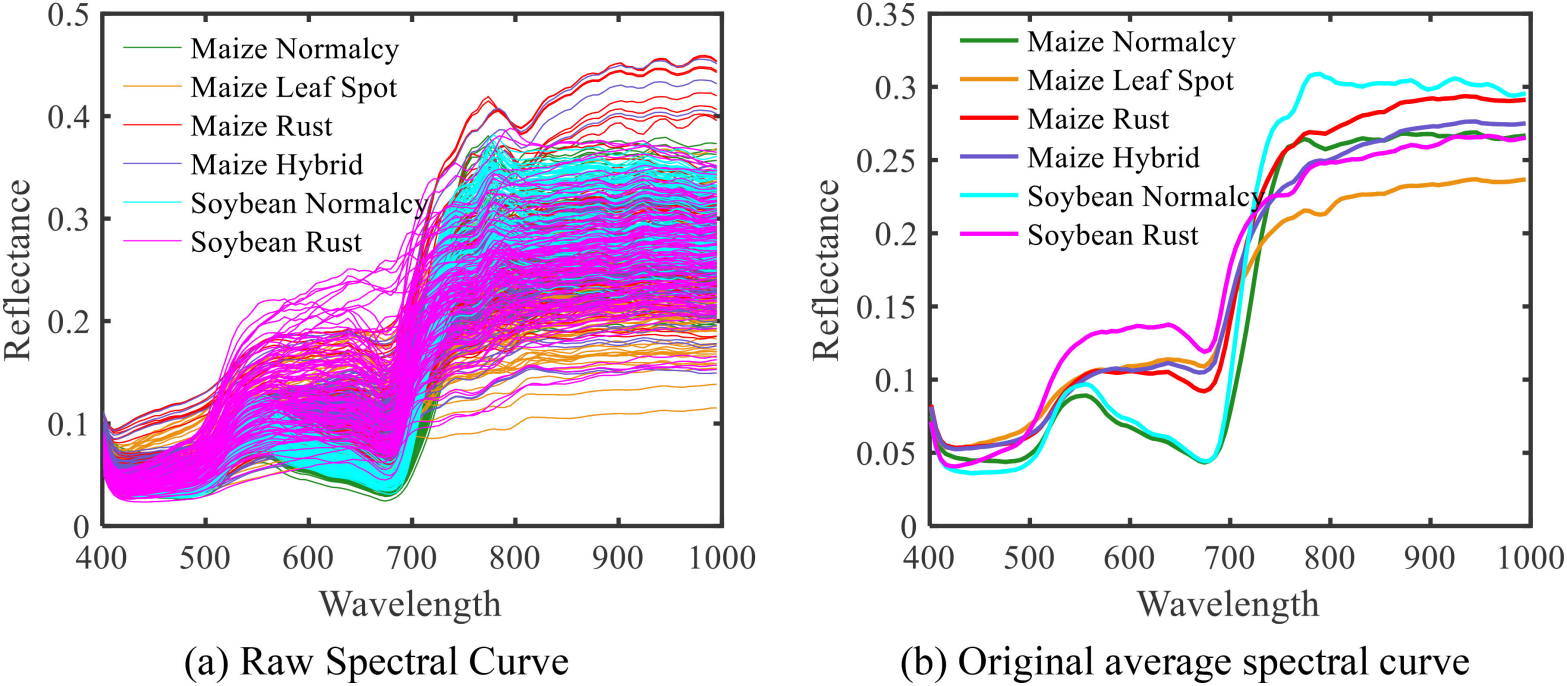
Spectrogram of maize-soybean diseased leaves. **(A)** Raw Spectral Curve; **(B)** Original average spectral curve.

Upon extracting regions of interest from the collected samples, we obtained the reflectance of the original spectral data depicting normal maize leaves, leaves with spots, leaves infected with rust, a combination of both diseases, as well as normal and rust-infected soybean leaves (refer to **Figure 7A**). Additionally, **Figure 7B** displays the average spectra of various maize and soybean samples. The research findings reveal that, across all leaf types, the reflectance undergoes a rapid increase within the 700-800 nm range, stabilizing beyond 800 nm. Significantly, noticeable troughs appear in the 550-700 nm range, with distinct variations in trough values among different leaf types. Spectral curves of normal maize and soybean leaves exhibit similar trends with roughly equivalent values in this range. In contrast, spectral data from various diseased leaves deviate significantly from the normal within this wavelength band, establishing a crucial foundation for subsequent classification efforts. Variations among the six leaf types are also apparent in the 700 nm to 1000 nm range, underscoring specific wavelength segments where maize and soybean leaves with different diseases demonstrate distinctions in their spectral information. This aids in constructing classification models utilizing their spectral information and provides essential conditions for further research on maize and soybean diseases.

**Figure 8:**
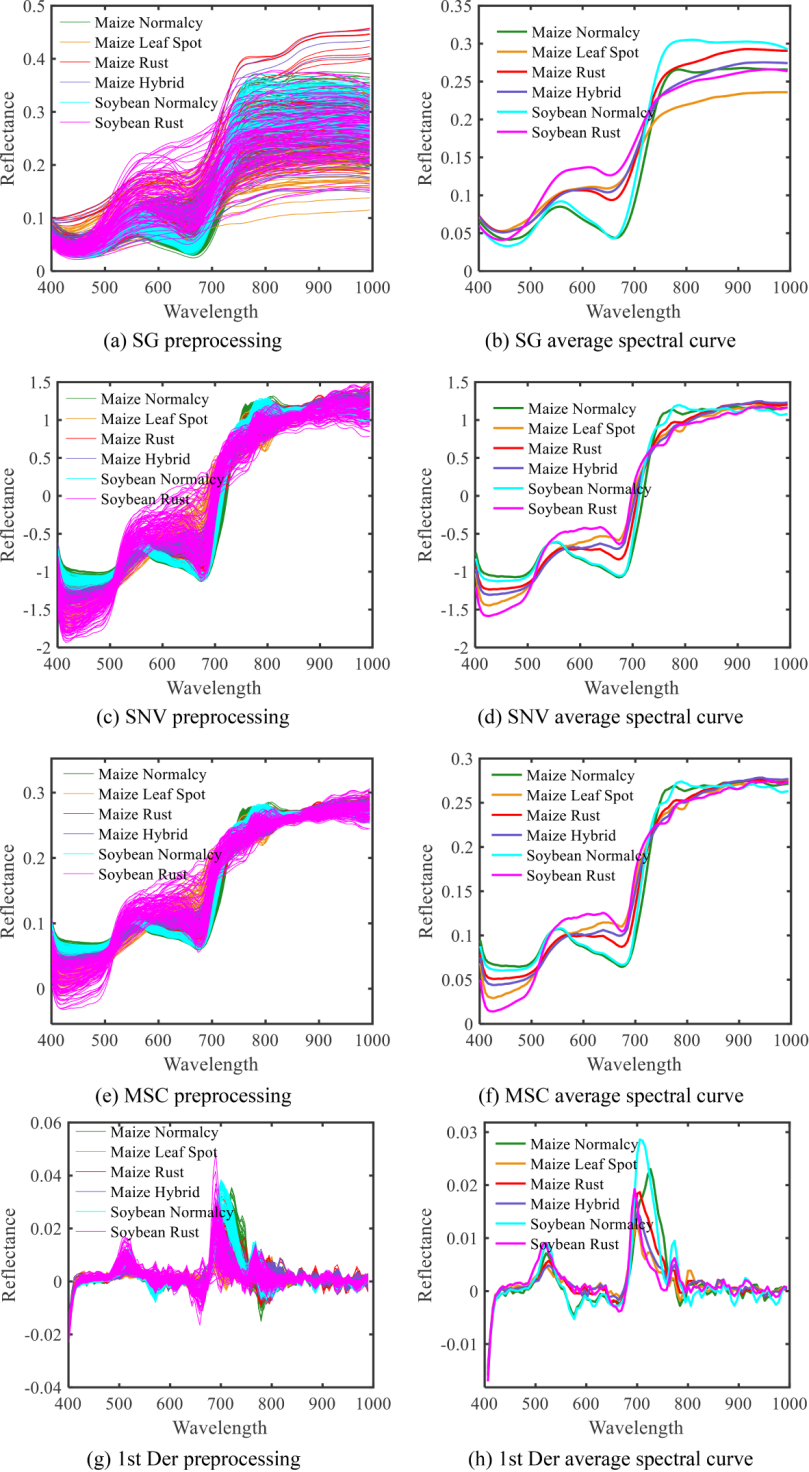
Spectral preprocessing map of diseased maize-soybean leaves. **(A)**SG processing; **(B)** SG average spectral curve; **(C)** SNV processing; **(D)** SNV average spectral curve; **(E)**MSC processing; **(F)** MSC average spectral curve; **(G)** 1 st Der processing; **(H)** 1 st Der average spectral curve.

The initial spectral data of maize and soybean underwent three preprocessing methods: SG, SNV and MSC, aimed at mitigating noise introduced during the spectral data collection process. **Figure 8** illustrates the preprocessing results. In comparison to the raw spectra, the SG-processed spectral curves appear smoother, indicating a relative reduction in the impact of noise. MSC and SNV methods effectively alleviate the scattering effects caused by non-uniform sample distribution and variable particle sizes, simultaneously addressing baseline drift and shifts induced by the collection environment. Spectral curves after SNV and MSC processing exhibit a more compact form, effectively reducing spectral differences caused by varying scattering levels, thereby enhancing the correlation within the spectral data. Furthermore, after first derivative (1st Der) processing, the spectral curve features become more prominent, alleviating baseline drift, eliminating background interference, and providing higher resolution.

#### 3.1.2 Comparison of preprocessing modelling results

To identify the optimal preprocessing method, spectral data processed through various techniques were employed as independent variables, with disease categories serving as the dependent variable. The training set comprised 450 samples, while the test set consisted of 150 samples, maintaining a ratio of 3:1. In this modeling the DBO algorithm dung beetle population size is 10 and the maximum number of iterations is 5. The optimized BiLSTM network parameters are set as shown in **Table 1**. Classification models, including SVM, BiLSTM, and DBO-BiLSTM, were established.

**Table 1:** DBO optimization of BiLSTM network parameter values.

Training parameter	Parameters selection	Training parameter	Parameters selection	Training parameter	Parameters selection
number of hidden units	158	mini-batch size	15	L2Regularization	0.0057
maximum training epochs	102	initial learning rate	0.03		

In this paper, two evaluation indexes are introduced, namely, Accuracy to judge the overall classification ability of the model and Recall to judge the classification ability of the model for a certain disease type, with Accuracy as the main index and Recall as the secondary index to judge the classification effect of the model, and their calculation formulas are as follows:

Accuracy: the proportion of all correctly predicted samples to the total sample.


(9) $$Accuracy=\frac{x_{1}+x_{2}+\cdot \cdot \cdot +x_{i}}{y_{1}+y_{2}+\cdot \cdot \cdot +y_{i}}\times 100\%=\frac{x}{y}\times 100\%$$

Recall: the proportion of samples predicted to be in a particular category as a percentage of such samples.


(10) $$Recall=\frac{x_{i}}{y_{i}}\times 100\%$$

where x and y denote the number of correctly predicted samples and the total number of all samples, respectively; i denotes the category; $x_{i}$ denotes the number of categories predicted to be i , which is actually the number of categories; and $y_{i}$ denotes the total number of samples in the i categories.

The classification results are shown in **Tables 2**–**4**. The data in the three tables show that DBO-BiLSTM outperforms SVM and BiLSTM in terms of classification accuracy. Compared to the other two models, DBO-BiLSTM consistently achieves higher classification accuracies for both the training set and the test set under different preprocessing methods. After 1st Der processing, the model performance exceeded the other three methods, so the 1st Der preprocessing method was chosen in this study considering the modelling effect and the classification accuracy of each disease. Also, the lower accuracy of the three classification models when constructed directly using preprocessed hyperspectral data emphasis the importance of feature wavelength extraction.

**Table 2:** SVM maize-soybean diseased leaf identification model based on different spectral preprocessing methods.

Model	Method	Training set classification recall/%						General accuracy		Test set classification recall/%					General accuracy
		Maize normalcy	Maize spot	Maize rust	Maize hybrid	Soybean normalcy	Soybean rust		Maize normalcy	Maize spot	maize rust	Maize hybrid	Soybean normalcy	Soybean rust	
SVM	SG	100.0	88.7	77.3	47.0	100.0	92.3	84.2	100.0	86.2	84.0	44.1	100.0	86.4	80.0
	SNV	97.4	84.0	86.7	61.8	98.7	94.9	87.8	100.0	72.0	68.0	68.8	100.0	100.0	83.3
	MSC	95.1	81.7	79.5	68.5	98.7	95.9	86.9	94.7	79.3	81.5	51.9	100.0	100.0	83.3
	1st Der	100.0	90.7	86.7	67.6	100.0	89.9	89.6	100.0	84.0	72.0	71.9	100.0	100.0	86.7

**Table 3:** BiLSTM maize-soybean diseased leaf identification model based on different spectral preprocessing methods.

Model	Method	Training set classification recall/%						General accuracy	Test set classification recall/%						General accuracy
		Maize normalcy	Maize spot	Maize rust	Maize hybrid	Soybean normalcy	Soybean rust		Maize normalcy	Maize spot	Maize rust	Maize hybrid	Soybean normalcy	Soybean rust	
BiLSTM	SG	98.6	88.8	59.2	83.5	100.0	84.9	86.0	100.0	75.0	58.6	76.2	100.0	81.5	82.0
	SNV	100.0	87.8	86.3	86.7	100.0	94.6	92.7	100.0	96.2	63.0	74.0	100.0	84.6	84.3
	MSC	100.0	86.8	77.0	87.2	100.0	91.8	90.4	100.0	95.8	61.5	71.8	100.0	88.9	85.0
	1st Der	100.0	90.3	92.5	67.1	100.0	96.1	91.1	100.0	85.7	90.0	63.0	100.0	75.0	85.3

**Table 4:** DBO-BiLSTM maize-soybean diseased leaf identification model based on different spectral preprocessing methods.

Model	Method	Training set classification recall/%						General accuracy	Test set classification recall/%						General accuracy
		Maize normalcy	Maize spot	Maize rust	Maize hybrid	Soybean normalcy	Soybean rust		Maize normalcy	Maize spot	Maize rust	Maize hybrid	Soybean normalcy	Soybean rust	
DBO- BiLSTM	SG	100.0	88.2	87.3	79.5	100.0	87.5	90.4	100.0	91.7	81.0	55.6	100.0	96.4	**87.3**
	SNV	100.0	81.9	83.3	82.9	100.0	91.5	90.0	100.0	78.6	92.9	70.8	100.0	88.9	**88.7**
	MSC	100.0	87.8	86.8	79.2	100.0	94.7	91.6	100.0	88.5	66.7	85.7	100.0	96.0	**89.3**
	**1st Der**	**100.0**	**94.0**	**92.4**	**89.5**	**100.0**	**98.6**	**95.8**	**100.0**	**84.8**	**95.2**	**87.5**	**100.0**	**86.2**	**91.3**

The Bold values mean the best results.

### 3.2 Spectral feature extraction

#### 3.2.1 CARS

**Figure 9:**
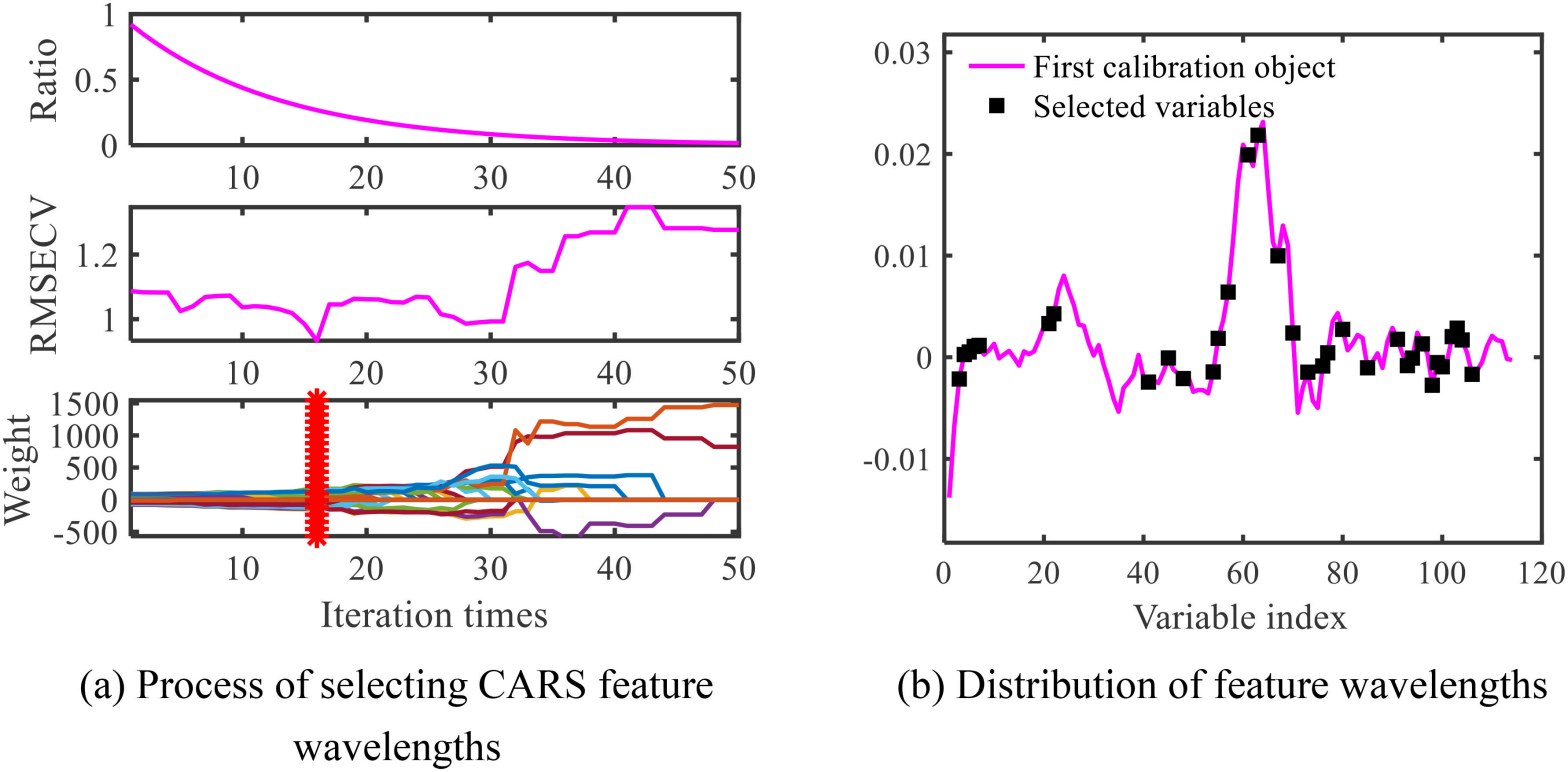
Wavelength map of CARS extraction characteristics for maize-soybean. **(A)** Process of selecting CARS feature wavelengths; **(B)** Distribution of feature wavelengths.

When applying the CARS algorithm for feature wavelength extraction on 1st Der preprocessed spectral data, 50 Monte Carlo sampling iterations were conducted to identify the iteration count with the minimum RMSECV value to determine the optimal feature wavelengths. The outcomes of the CARS algorithm are presented in **Figure 9**. In **Figure 9A**, the RMSECV value attains its minimum at the 16th iteration and subsequently exhibits fluctuations. This implies that wavelengths excluded after the 16th iteration contain significant valuable information. Therefore, at the 16th iteration, the optimal feature wavelength set was selected, consisting of 33 different wavelengths. The location information of these extracted feature wavelengths is illustrated in **Figure 9B**.

#### 3.2.2 SPA

**Figure 10:**
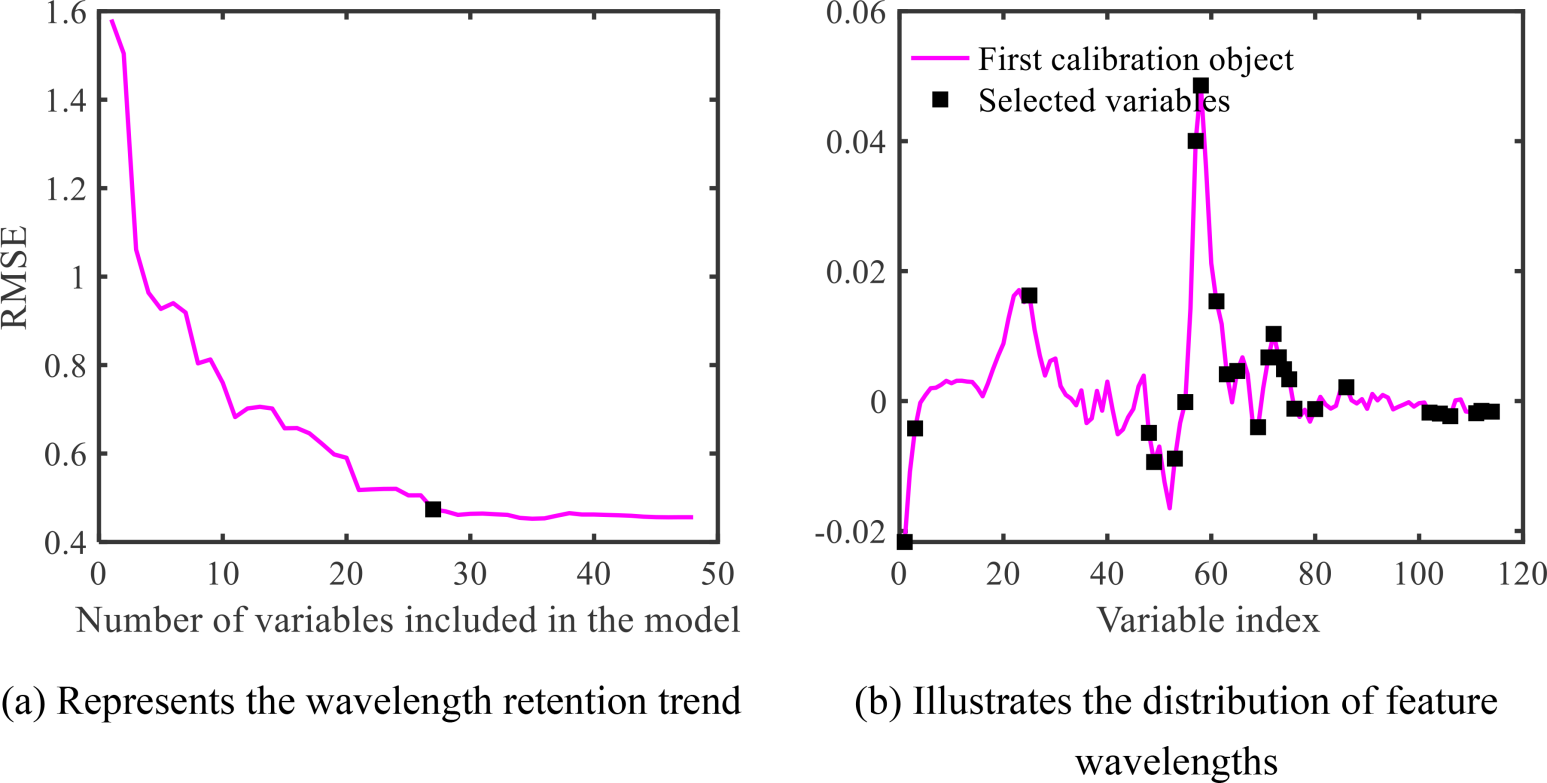
SPA extracted characteristic wavelength map of maize-soybean. **(A)** Represents the wavelength retention trend; **(B)** Illustrates the distribution of feature wavelengths.

In order to determine the optimal number of features, the SPA algorithm introduces the variable RMSE, through the size of this variable, to control the number of feature wavelengths that are most suitable for the 1st Der processed maize-soybean data, and if the RMSE reaches the lowest value, the selected feature wavelength at this time is the optimal feature wavelength. The results obtained through SPA are illustrated in **Figure 10**. It is observed that when the number of feature wavelengths is dataset to 27, the RMSE value approaches its minimum. As the number of feature wavelengths exceeds 27, the RMSE value does not exhibit significant changes. Therefore, 27 feature wavelengths are selected based on the final RMSE. The location information of the selected feature wavelengths is depicted in **Figure 10B**.

#### 3.2.3 PCA

**Figure 11:**
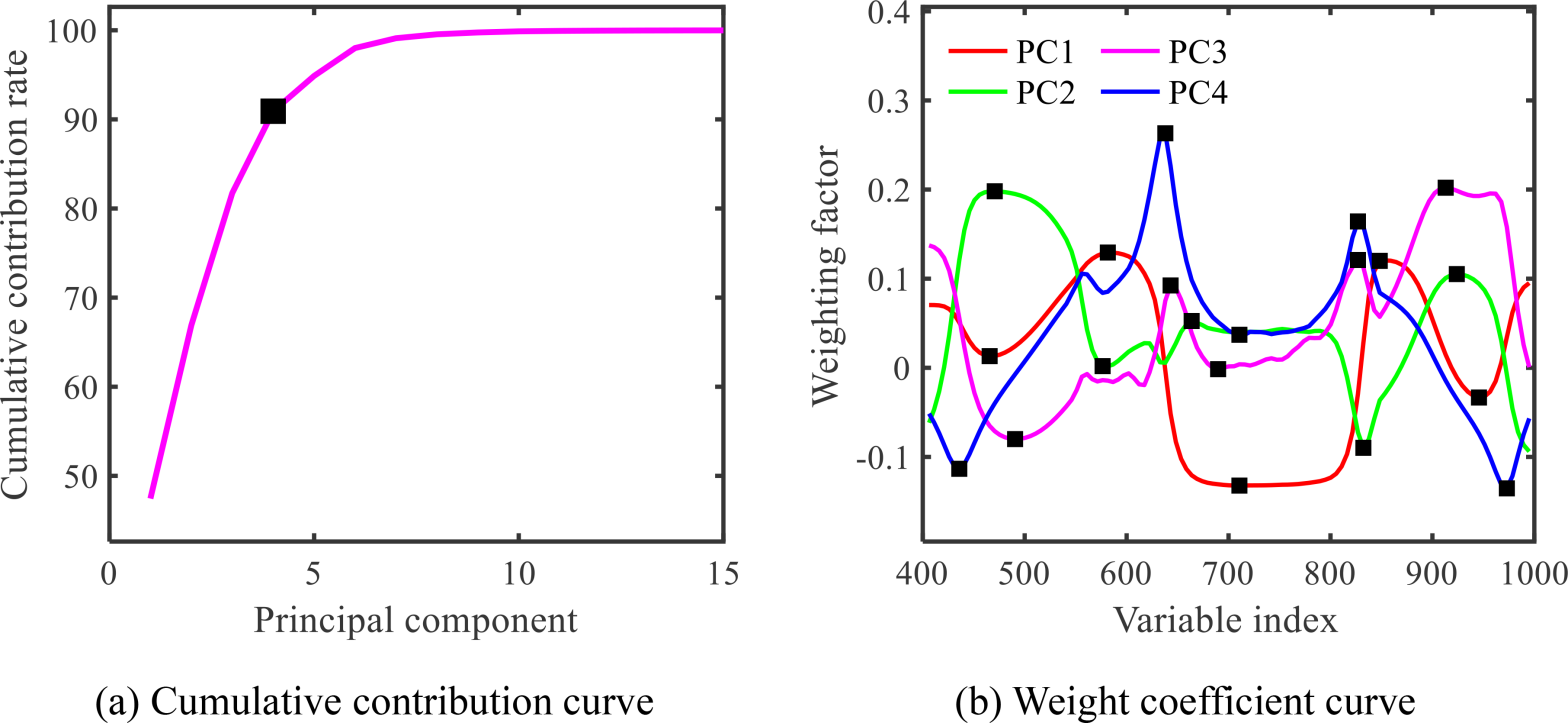
PCA extracted characteristic wavelength map of maize-soybean. **(A)** Cumulative contribution curve; **(B)** Weight coefficient curve.

Determining the number of principal components that can provide a more comprehensive overview of the information contained in the maize-soybean dataset after 1st Der processing is the first step in principal component analysis. This is achieved by ensuring that the cumulative contribution rate of these components reaches a relatively high value, typically not less than 85%. Subsequently, based on the weight coefficients of each wavelength in the principal components, wavelengths are selected at the peaks (or troughs) of the weight coefficient curve as feature wavelengths. The results obtained through PCA are shown in **Figure 11**. As depicted in **Figure 11A**, the cumulative contribution rate of the first 4 principal components exceeds 85%, reaching 90.9184%. Therefore, this study selected the first 4 principal components to represent the spectral information of maize and soybean leaves. Finally, 5 peaks are selected for each principal component, resulting in a total of 20 feature wavelengths. The distribution of the selected feature wavelengths is illustrated in **Figure 11B**.

### 3.3 Establishment and analysis of the classification model

To identify the most effective feature extraction method and the model with the best classification performance, SVM, BiLSTM, and DBO-BiLSTM classification models were established using a training set and a test set, maintaining a sample ratio of 3:1. This implies that the training set consists of 450 samples, while the test set comprises 150 samples. In this modeling the DBO algorithm dung beetle population size is 10 and the maximum number of iterations is 5. The optimized BiLSTM network parameters are set as shown in **Table 5**.

**Table 5:** DBO optimization parameter values.

Training parameter	Parameters selection	Training parameter	Parameters selection	Training parameter	Parameters selection
number of hidden units	98	mini-batch size	24	L2Regularization	0.0031
maximum training epochs	85	initial learning rate	0.007		

The classification results of the models are shown in **Tables 6**–**8**. Compared with the overall classification accuracies of the test set with only the 1st Der treatment in **Tables 2**–**4**, the accuracies in **Tables 6**–**8** were improved after feature extraction. This indicates that feature extraction plays an important role in improving the performance of disease classification in maize and soybean. From the point of view of different diseases, the test set of corn rust and mixed corn leaf rust had the lowest classification accuracy among the three models. Observation of **Figures 3C, D** reveals that both types of leaves are almost always covered by rust and are difficult to differentiate even with leaf spots, leading to challenging classification of these two types. On the contrary, maize and soybean were classified with 100% accuracy due to the significant difference between healthy and diseased leaves.

**Table 6:** SVM maize-soybean diseased leaf identification model based on different feature extraction methods.

Model	Method	Training set classification recall/%						General accuracy		Test set classification recall/%					General accuracy
		Maize normalcy	Maize spot	Maize rust	Maize hybrid	Soybean normalcy	Soybean rust		Maize normalcy	Maize spot	Maize rust	Maize hybrid	Soybean normalcy	Soybean rust	
SVM	CARS	100.0	91.7	85.7	83.6	100.0	98.6	93.6	100.0	92.9	76.7	74.1	100.0	93.5	88.0
	SPA	100.0	77.1	84.9	82.3	100.0	91.9	89.6	100.0	70.0	92.6	76.2	100.0	88.5	87.3
	PCA	96.1	84.0	73.2	62.8	98.7	91.7	84.4	95.8	84.0	72.4	54.5	100.0	82.1	81.3

**Table 7:** BiLSTM maize-soybean diseased leaf identification model based on different feature extraction methods.

Model	Method	Training set classification recall/%						General accuracy	Test set classification recall/%						General accuracy
		Maize normalcy	Maize spot	Maize rust	Maize hybrid	Soybean normalcy	Soybean rust		Maize normalcy	Maize spot	Maize rust	Maize hybrid	Soybean normalcy	Soybean rust	
BiLSTM	CARS	100.0	95.0	81.9	84.7	100.0	100.0	93.8	100.0	90.0	75.0	78.6	100.0	95.0	89.3
	SPA	100.0	80.3	89.3	85.0	100.0	97.4	92.0	100.0	72.4	76.0	85.0	100.0	95.5	88.0
	PCA	100.0	97.1	81.1	83.6	100.0	100.0	93.8	100.0	86.7	69.2	70.4	100.0	100.0	86.7

**Table 8:** DBO-BiLSTM maize-soybean diseased leaf identification model based on different feature extraction methods.

Model	Method	Training set classification recall/%						General accuracy	Test set classification recall/%						General accuracy
		Maize normalcy	Maize spot	Maize rust	Maize hybrid	Soybean normalcy	Soybean rust		Maize normalcy	Maize spot	Maize rust	Maize hybrid	Soybean normalcy	Soybean rust	
DBO- BiLSTM	**CARS**	**100.0**	**98.7**	**100.0**	**97.2**	**100.0**	**100.0**	**99.3**	**100.0**	**100.0**	**94.7**	**96.6**	**100.0**	**100.0**	**98.7**
	SPA	100.0	98.7	97.4	95.7	100.0	100.0	98.6	100.0	95.5	91.3	90.0	100.0	100.0	96.0
	PCA	100.0	94.7	96.3	90.1	100.0	100.0	96.9	100.0	100.0	94.7	72.4	100.0	100.0	94.0

The Bold values mean the best results.

**Figure 12:**
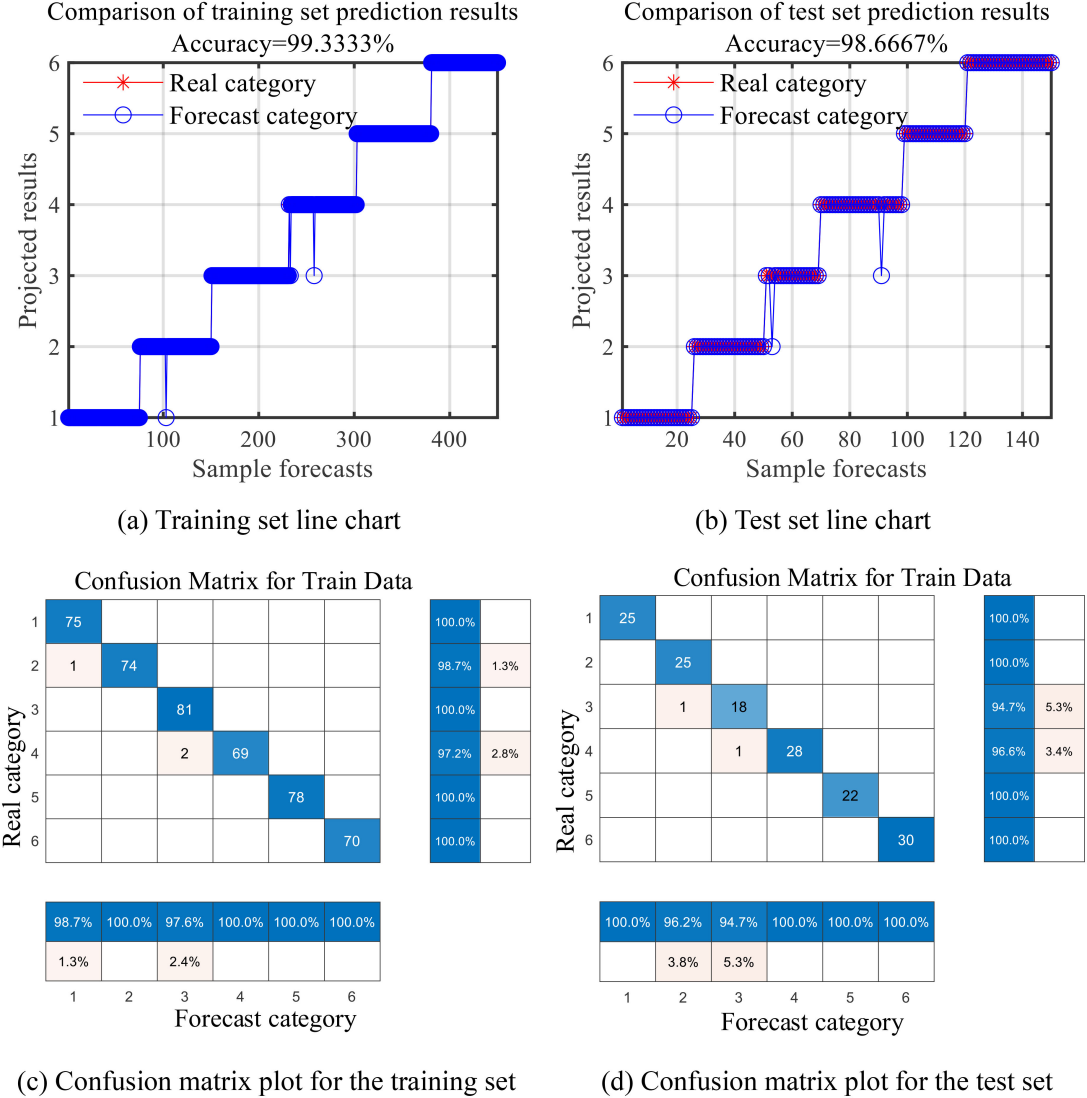
DBO-BiLSTM classification effect diagram (1, 2, 3, and 4 represent healthy, leaf spot, rust, and mixed leaf spot rust leaves for maize, and 5 and 6 represent healthy and rusted leaves for soybean). **(A)** Training set line chart; **(B)** Test set line chart; **(C)** Confusion matrix plot for the training set; **(D)** Confusion matrix plot for the test set.

The classification accuracy of the DBO-BiLSTM model is visually presented in **Figure 12**, showcasing its robust performance across different disease categories. These findings highlight the effectiveness of combining the CARS feature extraction method with the DBO-BiLSTM model and provide valuable insights for improving the accuracy of maize and soybean leaf disease discrimination.

## 4 Discussion

In this paper, after processing the hyperspectral data using four preprocessing methods: 1st Der, MSC, SNV, and SG, it can be seen through **Tables 2**, **3**, and **4** that there is a slight difference in the classification performance of the models in the hyperspectral data preprocessed by MSC and SNV, with the accuracy of the MSC test set being slightly better than that of the SNV. This advantage is attributed to the fact that the MSC is able to maximally retain the original spectral information associated with the disease type. After 1st Der processing, the model performance exceeded the other three methods, so 1st Der was chosen as the preprocessing method in this paper. In addition, SG preprocessing was the least effective, probably due to the fact that SG only smoothed the data. It can also be observed from the table that the classification performance of the mixed disease samples involving maize rust and rusty leaf spot was significantly lower than the other samples, indicating that the results were relatively unstable. This is mainly attributed to the/d fact that the surface of the spots on the mixed disease leaves was covered by rust, which negatively affected the classification of the model.

Among the different feature extraction methods, as shown in **Tables 6**, **7**, and **8**, the classification accuracy based on the PCA algorithm was lower than those based on the CARS algorithm and the SPA algorithm, indicating that the PCA algorithm was less effective in extracting maize and soybean disease features in this study. Notably, the CARS algorithm performed the best, highlighting the applicability of CARS in this study. **Figure 9** shows that CARS selects feature bands in a more dispersed manner, aiming to represent most of the information, thus reducing the loss of important information bands. In terms of model classification performance, **Tables 6** **-** **8** show that the DBO-BiLSTM model significantly outperforms SVM and BiLSTM. when combined with the CARS algorithm, the DBO-BiLSTM model achieves a classification accuracy of 98.7% on the test set, whereas the classification accuracy of SVM and BiLSTM is only 88.0% and 89.3%, respectively, which indicates a significant improvement in classification accuracy. This also highlights the strong optimization performance of the DBO algorithm in classification of the corn-soybean disease. Combining the DBO-BiLSTM model with the SPA and PCA feature extraction algorithms also resulted in higher classification performance than the other two models. This emphasis that the DBO-BiLSTM model outperforms the SVM and BiLSTM models when combining the different feature extraction methods, with classification accuracies of 98.7%, 96.0% and 94.0%, respectively.

## 5 Conclusion

This study focuses on maize leaf spot disease, rust disease, their combination, and soybean rust disease in an intercropping system of maize and soybean. Hyperspectral imaging technology was employed to collect hyperspectral data within the 400 to 1000 nm wavelength range. Through various preprocessing methods and models, significant results were achieved in the classification of maize and soybean samples.

1. Four preprocessing methods were applied to the raw spectral data: 1st Der, MSC, SNV, and SG. Modeling analysis determined that, the 1st Der preprocessing method exhibited the optimal processing performance.
2. CARS, SPA, and PCA were employed as feature extraction methods to extract feature wavelengths, resulting in 33, 27, and 20 feature wavelengths, respectively. As a result, the CARS feature extraction method demonstrated the most effective extraction performance.
3. Using the DBO optimization algorithm, five parameters of BiLSTM, were optimized to construct the DBO-BiLSTM neural network model, exhibiting significantly improved performance compared to SVM and BiLSTM models.
4. Among various model combinations, the 1st Der-CARS-DBO-BiLSTM model exhibits the best classification performance. This combination achieved a classification accuracy of 98.7% for the classification of maize and soybean diseases in the intercropping systems, providing a solid theoretical foundation and technical support for the accurate and non-destructive identification of various crops and diseases in intercropping systems.

The leaves collected in this study were from natural growing conditions, but the hyperspectral images were obtained in the laboratory after the leaves were harvested rather than *in situ*, which introduces some degree of idealized experimental conditions. In addition, due to the experimental conditions, this study only focused on several diseases in intercropped maize and soybean. Future research could further expand the experimental setting to include field collection of crop leaf hyperspectral images from field plantings and inclusion of additional crops and diseases, aiming to improve the utility of the model for high crop yields and quality.

## Data Availability

The raw data supporting the conclusions of this article will be made available by the authors, without undue reservation.
